# Clinical outcomes after one anastomosis gastric bypass versus sleeve gastrectomy in super-super-obese patients

**DOI:** 10.1007/s00464-021-08790-7

**Published:** 2021-10-26

**Authors:** Sophia M.-T. Schmitz, Patrick H. Alizai, Andreas Kroh, Sandra Schipper, Jonathan F. Brozat, Andreas Plamper, Ulf P. Neumann, Karl Rheinwalt, Tom F. Ulmer

**Affiliations:** 1 Department of General, Visceral- and Transplantation Surgery, RWTH Aachen University Hospital, Pauwelsstr. 30, 52074 Aachen, Germany; 2 Department of Gastroenterology, Digestive Diseases and Intensive Care Medicine, RWTH Aachen University Hospital, Pauwelsstr. 30, 52074 Aachen, Germany; 3 Department of Surgery, Maastricht University Medical Center, P. Debyelaan 25, 6229 HX Maastricht, The Netherlands; 4 Department of Bariatric, Metabolic and Plastic Surgery, St. Franziskus-Hospital, Schönsteinstr. 63, 50825 Cologne, Germany

**Keywords:** Bariatric surgery, Super-super-obese, SSO, One anastomosis gastric bypass, OAGB

## Abstract

**Background:**

Bariatric surgery in super-super-obese (SSO) patients remains a continuous challenge due to intraabdominal fat masses, higher liver volume and existing comorbidities. A convenient procedure in SSO patients is one anastomosis gastric bypass (OAGB). The aim of this study was to compare the outcome of SSO patients undergoing OAGB in comparison to laparoscopic sleeve gastrectomy (LSG).

**Methods:**

We retrospectively reviewed data from SSO patients who underwent OAGB and LSG in our institution between 2008 and 2020. Primary endpoints included percentage total body weight loss and percentage BMI loss at 12, 24, and 36 months after the operation. Secondary endpoints were perioperative complications, procedure length, length of hospital stay and outcome of comorbidities.

**Results:**

243 patients were included in this study. 93 patients underwent LSG and 150 underwent OAGB. At any of the time points evaluated, weight loss in patients after OAGB was greater than in LSG patients, while procedure length was significantly shorter for OAGB than LSG (81.4 vs. 92.1 min, *p*-value < 0.001). Additionally, mean length of hospital stay was shorter in the OAGB group (3.4 vs. 4.5 days, *p*-value < 0.001). There were more severe complications (Clavien-Dindo ≥ 3a) in the LSG group (11.8% vs 2.7%, *p*-value = 0.005).

**Conclusion:**

In this retrospective analysis, OAGB was superior to LSG in terms of weight loss in SSO patients. Procedure length and hospital stay were shorter after OAGB in comparison to LSG and there were fewer severe complications. OAGB can therefore be regarded a safe and effective treatment modality for SSO patients.

Bariatric and metabolic surgery meets wide acceptance with an acceptable perioperative risk profile even in patients with higher perioperative risk profiles. Especially super-super obese (SSO) patients with a Body-Mass-Index (BMI) > 60 kg/m^2^ are prone to operation risks and morbidity [1–3], caused by difficulties in exposure, a fatty liver, tension on the surgical instruments and co-existing comorbidities [4–6]. Therefore, operation time, length of hospital stay, and complication rates have been described to be higher in SSO patients in comparison to patients with a BMI < 60 kg/m^2^ [1, 3].

Among the possible operative approaches in SSO patients is laparoscopic sleeve gastrectomy (LSG), as it is safe and easily feasible with acceptable weight reduction outcomes [5, 7, 8]. Furthermore, following an initial weight loss, there is the possibility of a two-stage procedure, adding laparoscopic Roux-en-Y Gastric Bypass (RYGB) or other options like Single Anastomosis Duodeno-ileal bypass with Sleeve Gastrectomy (SADI-S), Biliopancreatic Diversion with Duodenal Switch (BPD-DS) or One Anastomosis Gastric Bypass (OAGB) as a second step [5, 9]. While weight loss is superior in SADI-S or BPD-DS in comparison to RYGB after failed SG, these methods are technically more challenging and might imply higher risks for complications [10, 11]. RYGB has also been described as a primary procedure in SSO patients, but complication rates and length of stay usually have been reported to be significantly higher than after LSG [5, 7, 12]. A suitable approach in SSO patients is the One Anastomosis Gastric Bypass (OAGB), that is comparable to LSG in safety and might yield superior results regarding weight loss and remission of comorbidities [13–20]. OAGB has been reported to be safe in SSO patients with a BMI > 60 kg/m^2^ [13, 21] and seems to be even superior to RYGB in terms of reduced complications [22] and improved weight loss [21]. While data on the comparison of OAGB and LSG in patients with morbid obesity is still scarce [18, 19, 23–25], it is almost absent for SSO patients with the exception of a small retrospective cohort analysis [26].

The aim of this study was therefore to retrospectively compare OAGB and LSG in terms of weight loss and short- to mid-term outcomes in a large cohort of SSO patients.

## Methods

SSO patients with a BMI > 60 kg/m^2^ that underwent either LSG or OAGB between 2008 and 2020 were identified from a prospective surgical database.

The decision on the operative approach was taken depending on the patient’s type of individual fat distribution, small bowel mobility, medications, individual preferences and bowel habits. Patients with a history of gastroesophageal reflux disease were clearly orientated towards OAGB. Routinely all patients signed informed consent for both procedures, as the final decision of procedure was often dependent on the intraoperative conditions. Informed consent included the possible need to perform a two-stage surgery after LSG. All procedures were performed according to standardized operation techniques by a single bariatric surgeon experienced in both procedures.

All patients provided written consent to anonymized data registration in the national bariatric data base as well as statistical work-up. Ethical approval was not required due to the retrospective character of this study and the analysis of entirely pseudonymised data regarding well-established procedures. All procedures performed in studies were in accordance with the 1964 Helsinki declaration and its later amendments.

Demographic characteristics, BMI, weight, operation characteristics, as well as comorbidities and late complications were recorded prior to operation and at 3, 6, 9, 12, 24, and 36 months. Weight loss was reported as percent total body weight loss (%TBWL) and percent BMI loss (%BMIL). Postoperative complications were classified according to the Clavien-Dindo Classification during the hospital stay [27]. A Clavien-Dindo Score of 3a or higher was classified as a severe (major) complication. Partial and full remission of co-morbidities were evaluated according to the simplified Buchwald-criteria at the time points mentioned above [28]. The classification of insufficient weight loss and relevant weight regain was performed according to the criteria established by Reinhold [29].

## Operation characteristics

In LSG, four 12 mm-trocars and optionally an additional 5 mm-trocar were placed under videoendoscopic control in the upper abdomen. Capnoperitoneum with 15 mm Mercury pressure was applied, in cases with insufficient working space this pressure became elevated up to 20 mm Mercury. With the patient in Anti-Trendelenburg-position and left lobe of the liver retracted, the greater curvature of the stomach was dissected with ultrasonic scissors (Harmonic®, Johnson&Johnson) from 5 cm above the pylorus to the angle of His and the left crus. The gastric fundus was mobilized from the retroperitoneum as completely as possible. After introducing a 42 F calibration tube, the vertical dissection started distally with a black 60 mm cartridge using either mechanical or powered stapling device (Echelon®, Johnson&Johnson). The further dissection until 1.5 cm lateral to the angle of His was performed using green, yellow and then blue cartridges, thus respecting the decreasing thickness of the proximal gastric wall. The proximal 6 cm of the staple line were oversewn by running absorbable suture 2–0. Cross-sections of the staple-line and points of oozing were reinforced with metal clips. Routine leak test was performed. The specimen was retracted via the enlarged subcostal trocar on the left side using a specimen bag. This site was closed by a videoendoscopically applied full-thickness stitch with Vicryl® 1. Routinely, a subcostal drain was placed.

In OAGB, routinely four 12 mm-trocars and one 5 mm-trocar were applied. With the surgeon on the right side of the patient and optional retraction of the left lobe of the liver the procedure started with dissection of supragastric adhesions lateral from the angle of His towards the upper splenic pole. Then the proximal antrum became partially dissected starting from the minor curvature with a black 60 mm-cartridge (Echelon® mechanical or powered, Johnson&Johnson). This was followed by retrogastric dissection of adhesions and vertical complete dissection of the stomach parallel to a intraluminal 30F-calibration tube. For this procedure, usually four to five 60 mm cartridges with declining staple-height were used, forming a long and narrow pouch of 16 to 22 cm of length. A tension-free antecolic four to five cm long end-to-side-gastrojejunostomy was created after measuring a usually 250 cm biliopancreatic limb beginning at the ligament of Treitz. The ventral defect at the anastomosis was closed manually with 2-layers full-thickness running suture with absorbable thread 2–0. The large Petersen space was not closed. Routine blue test of pouch and anastomosis was performed, followed by clipping of cross-sections and oozing points of the staple line and subhepatic placement of a silicone drain.

### Statistical analysis

Data are expressed as mean (Standard error of mean, SEM) unless otherwise indicated. Independent *T*-Test and One-way ANOVA were used to compare means between groups. Chi-square (χ^2^) and Fisher’s exact test were used for comparison of categorical data. Statistical analysis was performed with IBM SPSS v18 and GraphPad Prism v7. A *p*-value < 0.05 was considered statistically significant.

## Results

### Basic demographics

A total of 243 patients (165 female, 68%) with a mean BMI of 65.2 kg/m^2^ (range 60.0–87.1 kg/m^2^) were included in this study. OAGB was performed in 150 and LSG in 93 patients. Mean BMI was 67 kg/m^2^ in the LSG group and 64 kg/m^2^ in the OAGB group (*p*-value < 0.001). Apart from coronary heart disease (LSG 12.9% vs. OAGB 5.3%, *p*-value = 0.037), comorbidities were comparable between the groups (Table 1).

**Table 1:** Demographic characteristics and comorbidities of patients undergoing OAGB and LSG

	Operative technique				*p*-value
	LSG (*n* = 93)		OAGB (*n* = 150)		
	*n* (%)	Mean (SEM)	*n* (%)	Mean (SEM)	
Sex					
Female	54 (58.1%)		112 (74.5%)		**0.008**
Age		41.57 (1.07)		39.11 (0.9)	0.646
Weight		194.7 (2.9)		183.7 (1.8)	**0.006**
BMI		66.91 (0.6)		64.14 (0.3)	** < 0.001**
Sleep apnea	69 (74.2%)		113 (75.3%)		0.444
T2DM	42 (45.7%)		51 (34.0%)		0.07
Orthopedic comorbidities	91 (97.8%)		142 (94.7%)		0.225
CHD	12 (12.9%)		8 (5.3%)		**0.037**
Hypertension	69 (74.2%)		98 (65.3%)		0.148

Bold values indicate statistical significance

*BMI* body mass index, *CHD* coronary heart disease, *LSG* laparoscopic sleeve gastrectomy, *OAGB* one anastomosis gastric bypass, *SEM* standard error of the mean, *T2DM* type 2 diabetes mellitus

### Operation characteristics and postoperative outcomes

Mean operating time was significantly shorter in the OAGB group (81.4 vs. 92.1 min, *p*-value < 0.001). Mean hospital stay was also significantly shorter in the OAGB group (3.4 days vs. 4.5 days, *p*-value < 0.001). Major postoperative complications (Clavien-Dindo-Score ≥ 3a) occurred more often after LSG in comparison to OAGB (11.8% vs. 2.7%, *p*-value = 0.005). Conversion and mortality rate were zero in both groups. Occurrence of gastrointestinal ulcers was higher in the OAGB group when compared to the LSG group (7.3% vs. 1%, *p*-value = 0.033). Postoperative dumping, reflux and malnutrition were observed rarely and showed no statistically significant differences between groups. See Table 2 for a comparison of the operative outcomes in the two groups.

**Table 2:** Characteristics of operation and postoperative course in patients undergoing OAGB and LSG

	Operative technique				*p*-value
	LSG (*n* = 93)		OAGB (*n* = 150)		
	Mean (SEM)	*n* (%)	Mean (SEM)	*n* (%)	
Operation characteristics and early postoperative complications					
Operating Time	92.08 (3.1)		81.36 (1.6)		** < 0.001**
Conversion		0 (0%)		0 (0%)	
Mortality		0 (0%)		0 (0%)	
Length of Hospital Stay	4.53 (0.2)		3.44 (0.1)		** < 0.001**
Clavien-Dindo Complication Score					
≤ 3		82 (88.2%)		146 (97.3%)	**0.005**
≥ 3		11 (11.8%)		4 (2.7%)	
Late postoperative complication					
Malnutrition		14 (30.4%)		32 (69.6%)	0.225
Insufficient weight loss/weight regain		28 (66.7%)		14 (33.3%)	** < 0.001**
Ulcer		1 (1%)		11 (7.3%)	**0.033**
Dumping		0 (0%)		3 (2%)	0.288
Reflux		10 (10.8%)		9 (6.0%)	0.180

Bold values indicate statistical significance

*LSG* laparoscopic sleeve gastrectomy, *OAGB* one anastomosis gastric bypass, *SEM* standard error of the mean, *T2DM* type 2 diabetes mellitus

### Weight loss

**Fig. 1:**
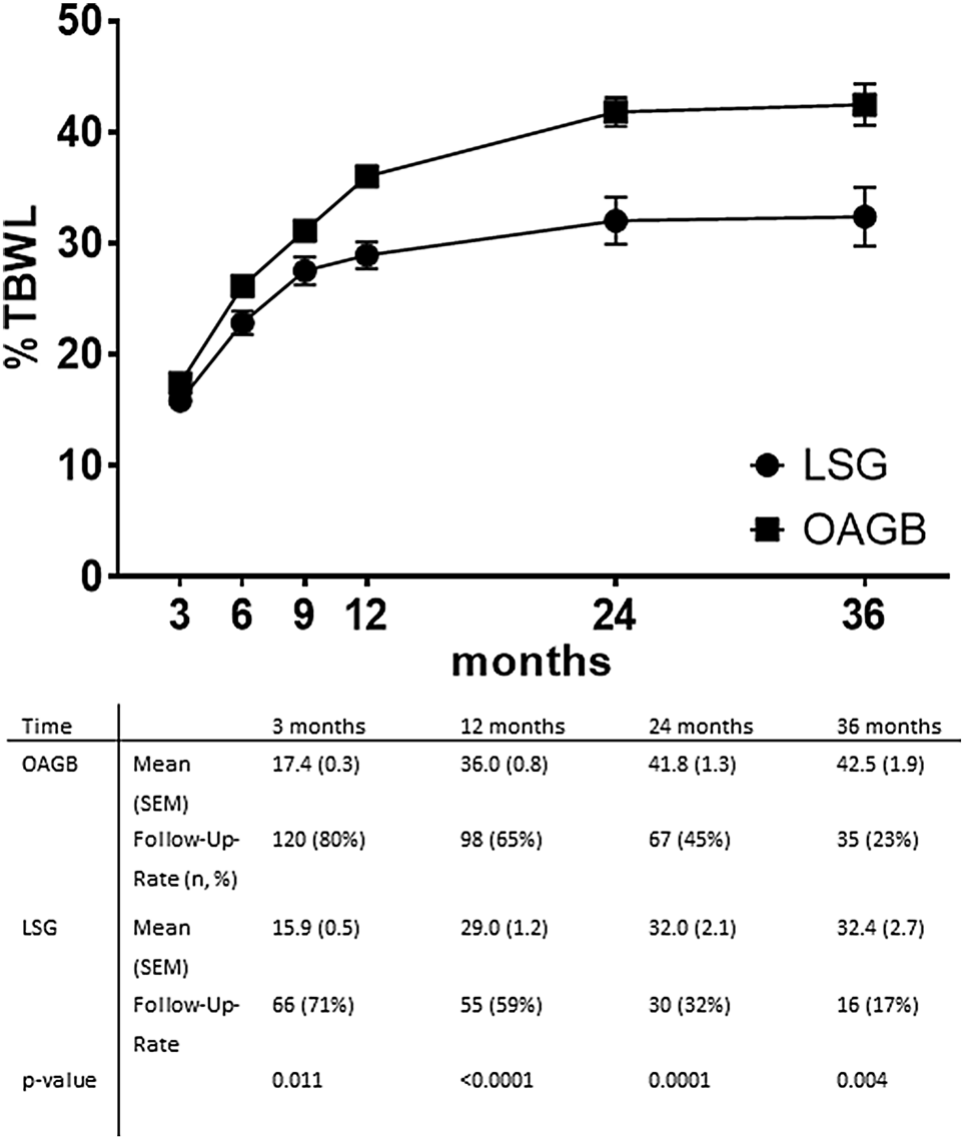
Weight loss outcomes in patients undergoing OAGB and LSG; *LSG* laparoscopic sleeve gastrectomy, *OAGB* one anastomosis gastric bypass, *TBWL* total body weight loss; there was a significant difference in TBWL at all time points

**Fig. 2:**
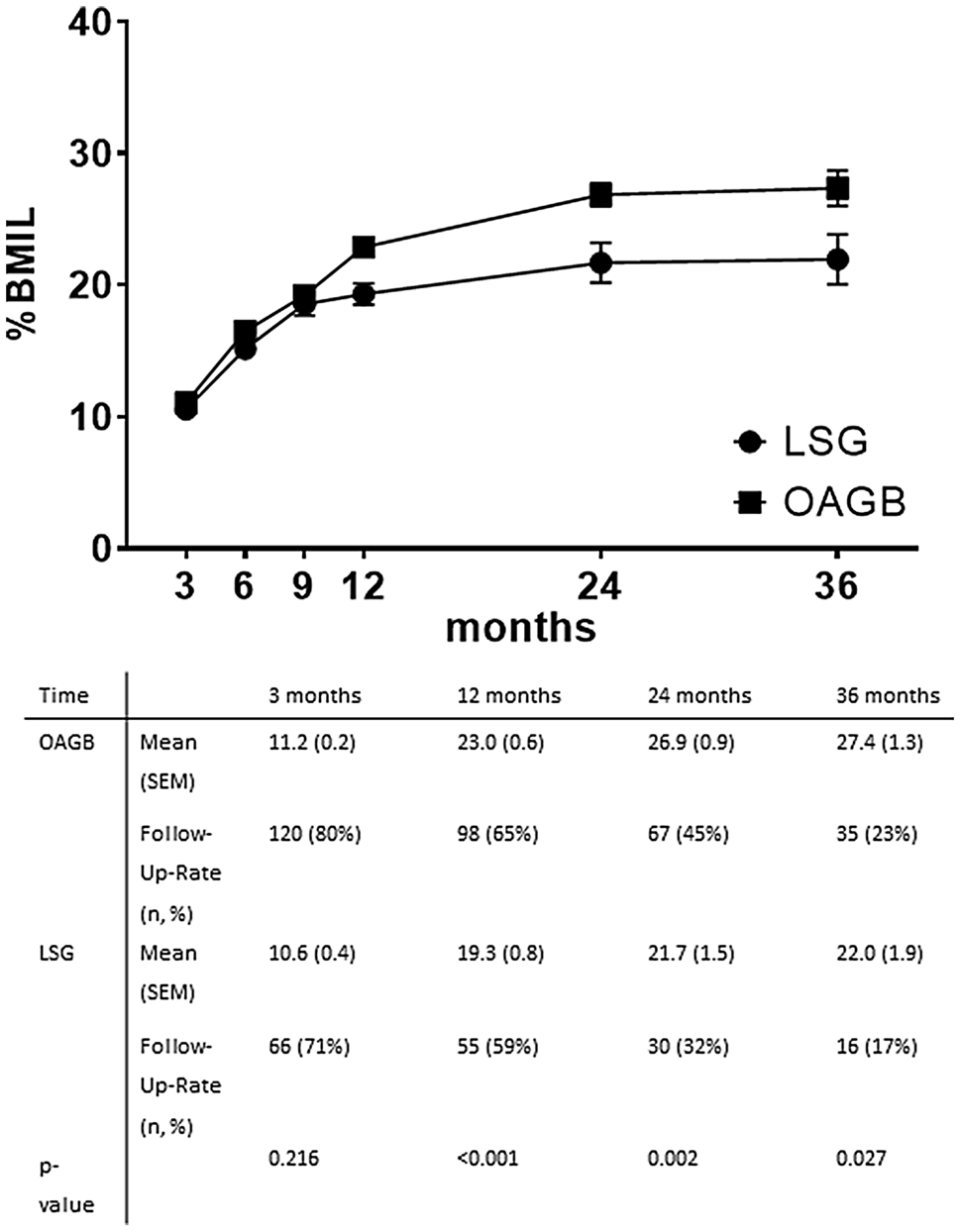
Weight loss outcomes in patients undergoing OAGB and LSG; *LSG* laparoscopic sleeve gastrectomy, *OAGB* one anastomosis gastric bypass, *%BMIL* percent BMI Loss

The follow-up-rate after 12 months was 65% in OAGB and 59% in LSG, after 24 months 45 and 32%, resp., and after 36 months 23 and 17%, resp. Following OAGB, %TBWL and %BMIL were significantly higher compared with LSG at all time points with the exception of %BMIL after three months (Figs. 1, 2). In comparison to OAGB, significantly more patients that underwent LSG suffered from insufficient weight loss or weight regain after LSG (14 vs. 28 patients, *p*-value < 0.001).

### Comorbidities

**Fig. 3:**
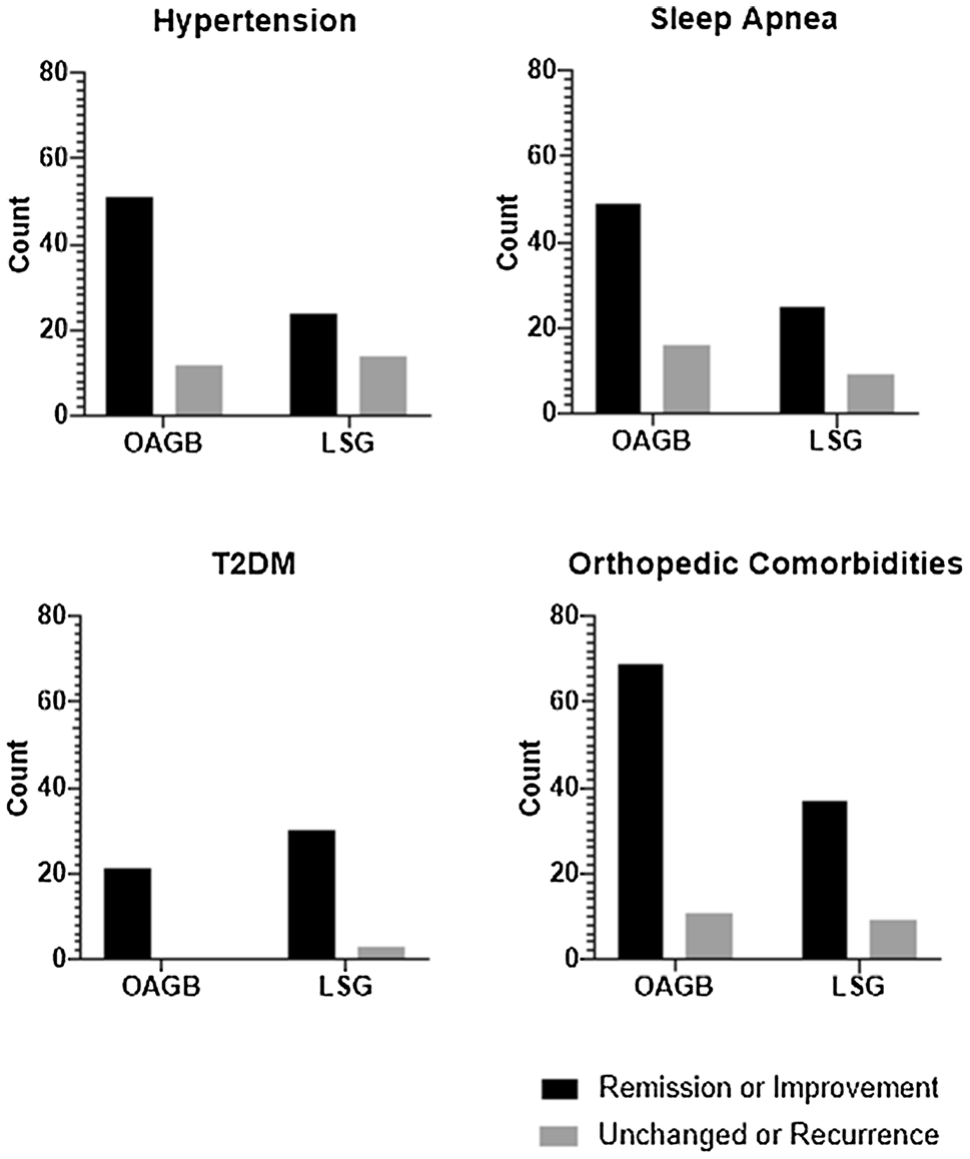
Comorbidity outcomes after 12 months of patients undergoing OAGB and LSG; *LSG* laparoscopic sleeve gastrectomy, *OAGB* one anastomosis gastric bypass, *SEM* standard error of the mean, *T2DM* type 2 diabetes mellitus; χ^2^-Test revealed a significant difference for remission of Hypertension between OAGB and LSG (*p*-value 0.048), for other comorbidities differences were not significant

There was no difference in remission or improvement of comorbidities (T2DM, sleep apnea, orthopedic comorbidities) with the exception of arterial hypertension, where remission rate was higher after OAGB than after LSG after 12 months (*p*-value = 0.048; Fig. 3).

## Discussion

The management of SSO patients is a persistent surgical challenge due to difficult intraoperative conditions and associated increase in perioperative risks. The ideal operative approach in these patients remains a subject of intense discussions. Up to now, most studies concerning bariatric surgery in SSO patients focussed on LSG and RYGB [1, 5–7, 12, 30]. As OAGB is performed less frequently in comparison to LSG and RYGB, evidence on patient-oriented outcomes following OAGB remains scarce [13, 26]. Moreover, most publications concerning results of OAGB focus on cohorts of patients with a wide range BMI-levels and do not focus exclusively on SSO patients [14–20, 22, 23, 25]. Parmar et al. compared RYGB with OAGB in SSO patients [21] and Singla et al. compared LSG with OAGB in patients with a BMI > 50 kg/m^2^ [24]. A review of Parmar et al. focused on OAGB versus LSG and RYGB in patients with a BMI > 50 kg/m^2^ [31]. Peraglie et al. examined a cohort of SSO patients receiving OAGB in a single center study, showing effectiveness and safety of this procedure in SSO patients [13]. In summary, both the underrepresentation of the procedure and the rareness of SSO patients add to most published studies being underpowered.

In this study, the outcome of 243 SSO patients was evaluated and OAGB resulted in superior weight loss in comparison to LSG while requiring shorter operation times and resulting in shorter lengths of hospital stay. Of interest, 28 patients in the LSG group compared to 14 patients in the OAGB group suffered from insufficient weight loss or weight regain after the operation, favoring possible superior short- to mid-term effects of OAGB in comparison to LSG in SSO patients.

Adding to this possible superiority, other publications including morbidly obese patients similarly report on higher weight loss after OAGB in comparison to LSG [18, 24, 32]. Admittedly, Kular et al*.* have reported a similar %EWL in patients after OAGB compared to LSG. However, these patients had a mean BMI of only 44 kg/m^2^ (OAGB) and 42 kg/m^2^ (LSG) and the results therefore seem barely comparable to our study [23]. In SSO patients, Parmar et al. showed superior weight loss of OAGB in comparison to RYGB [21].

Procedure length was significantly shorter for OAGB in comparison to LSG in our study (81.4 vs. 92.1 min, *p*-value < 0.001). There is divergent data on operation time for both procedures. A recent meta-analysis found no difference between LSG and OAGB in patients with a mean BMI of 41.8 kg/m^2^ and 40.8 kg/m^2^, respectively [18]. In general, operation time for bariatric procedures in SSO patients is longer than in non-SSO patients [1, 21]. In contrast to our study, Madhok et al. found significantly shorter operation times for LSG (75 min) in comparison toOAGB (92 min) in SSO patients [26]. These contradicting findings might partially be explainable by the higher BMI of our LSG patients at the time of the operation. Furthermore, over-sewing of staple lines and the need of specimen extraction might add to a longer operation time in LSG patients.

Postoperative complication rates have been reported in 15.1% (RYBG) and 4.8% (LSG) in SSO patients [12], while complication rates in OAGB generally have been reported to be less than 3% in patients with BMI > 50 kg/m^2^ [31]. In our study, we report a rate of severe complications (Clavien-Dindo ≥ 3a) in 11.8% (LSG) compared to 2.7% (OAGB) for SSO patients. In a large meta-analysis, the risk of postoperative leakage was higher after LSG compared to OAGB, while the risk of malnutrition and ulcers was increased after OAGB [18]. While we can confirm these findings in terms of a higher perioperative complication rate after LSG and higher risk of ulcers after OAGB, we did not find an increased rate of reported deficiencies for albumin, iron, calcium or vitamins after OAGB.

Another noteworthy finding is the higher rate of remission or improvement of arterial hypertension after OAGB, while for other comorbidities the differences was not significant. Other studies report no differences in resolution of comorbidities [25].

Inherent to its retrospective design, the present study is limited by sample size and study period. The follow-up rate at three years is exceptionally low and has to be considered carefully. However, we report higher follow-up rates than other studies on the same subject report [12]. Second, due to the fact that the final decision on which procedure to perform was made intraoperatively there might have been a tendency to perform LSG in challenging cases. Notwithstanding these limitations, this study is to our knowledge the largest one so far to compare OAGB and LSG in SSO patients, including therefore manifold clinical implications.

## Conclusion

We found significantly higher weight loss, lower complication rate, and similar remission of comorbidities in OAGB compared to LSG in SSO patients. Additionally, both procedure length and length of hospital stay were shorter after OAGB. Taken into account the limitations of this retrospective analysis, OAGB can be considered a safe and effective option in the treatment of SSO patients and can possibly even be considered superior to LSG in these patients.
